# Episodes of fever in neutropenia in pediatric patients with cancer in Bern, Switzerland, 1993–2012

**DOI:** 10.1038/sdata.2018.304

**Published:** 2019-01-15

**Authors:** Maxime G. Zermatten, Christa Koenig, Annina von Allmen, Philipp Agyeman, Roland A. Ammann

**Affiliations:** 1 Division of Pediatric Hematology/Oncology, Department of Pediatrics, Inselspital, Bern University Hospital, University of Bern, Bern, Switzerland; 2 Centre for Reviews and Dissemination, University of York, York, UK; 3 Department of Pediatrics, Inselspital, Bern University Hospital, University of Bern, Bern, Switzerland

**Keywords:** Fever, Outcomes research, Paediatric cancer

## Abstract

Fever in neutropenia (FN) is the most frequent potentially life threatening complication of chemotherapy for cancer. Prediction of the risk to develop complications, integrated into clinical decision rules, would allow for risk-stratified treatment of FN. This retrospective, single center cohort study in pediatric patients diagnosed with cancer before 17 years, covered two decades, 1993 to 2012. In total, 703 FN episodes in 291 patients with chemotherapy (maximum per patient, 9) were reported here. Twenty-nine characteristics of FN were collected: 6 were patient- and cancer-related, 8 were characteristics of history, 8 of clinical examination, and 7 laboratory results in peripheral blood, all known at FN diagnosis. In total 28 FN outcomes were assessed: 8 described treatment of FN, 6 described microbiologically defined infections (MDI), 4 clinically defined infections, 4 were additional clinical composite outcomes, and 6 outcomes were related to discharge. These data can mainly be used to study FN characteristics and their association with outcomes over time and between centers, and for derivation and external validation of clinical decision rules.

## Background & Summary

Fever in neutropenia (FN) is the most common potentially lethal complication of chemotherapy for cancer^1,2^. About half of the children treated with chemotherapy for cancer develop at least one FN episode^3,4^. In the 1970s up to 30% of children with FN died^1,5^. The introduction of routine emergency hospitalization and empirical administration of intravenous broad-spectrum antibiotics reduced mortality to around 1%^2,6^.

However, a bacterial infection is detected in a minority of children with FN. This implies overtreatment in the majority of children with FN^7,8^. Such overtreatment has important negative implications on both societal and individual level. These include costs both due to antibiotics and to hospitalization^9^, promotion of bacterial resistance, risk of nosocomial infections, and finally reduced quality of life of patients and their families during hospitalization^10^.

These observations led to the development and wide application of clinical decision rules (CDRs) predicting the risk of relevant complications in adults with FN^11^. The implementation of CDRs, and correspondingly of risk adapted treatment of FN, is actually recommended as well in pediatric oncology, but only rarely used in practice^12,13^. Currently, there is no consensus on which of the many published CDRs should be used for FN in pediatric oncology because the methodological quality of most CDRs is not optimal^14^, and none of these rules has been found to perform sufficiently well when externally validated^15–17^ with only one exception^8^ described very recently^18^. Differences in the populations studied^19^, in local management of FN and definitions of outcome criteria^20^, or missing clinical information in different datasets hamper even individual patient data meta-analysis^21,22^. Changes of clinical characteristics over time, and even of their associations with outcomes, may explain a further part of the insufficient predictive performance of these CDRs when validated externally.

This study aimed to collect long-term data on 29 predefined characteristics in pediatric patients at diagnosis of FN potentially associated with two main FN outcomes, bacteremia and severe bacterial infection (SBI). Of these characteristics 6 were patient- and cancer-related, 8 were characteristics of history, 8 of clinical examination, and 7 laboratory results in peripheral blood, all known at FN diagnosis. A total of 28 FN outcomes were assessed: 8 described treatment of FN, 6 described microbiologically defined infections (MDI), 4 clinically defined infections, 4 were additional clinical composite outcomes, and 6 outcomes were related to discharge.

The clinical motivation of this study was to generate data for the development and external validation of CDRs in pediatric FN, finally leading to evidence-based risk-stratified empirical treatment of FN in these patients.

This study was designed as a retrospective, single center cohort study in pediatric patients diagnosed with cancer before 17 years at the Division of Pediatric Hematology/Oncology, Department of Pediatrics, Inselspital, Bern University Hospital, University of Bern, Switzerland, covering two decades, 1993 to 2012. This hospital provides tertiary care for a population of roughly one million inhabitants. The Division of Pediatric Hematology/Oncology unit has an inpatient unit with 8 beds, plus a large outpatient unit. It treats around 40 newly diagnosed pediatric patients with all kind of malignancies per year. Besides, it performs myeloablative chemotherapy followed by autologous stem cell transplantation for the majority of Switzerland, covering around 6 million inhabitants.

All clinical information was directly extracted from patient charts.

**Figure 1:**
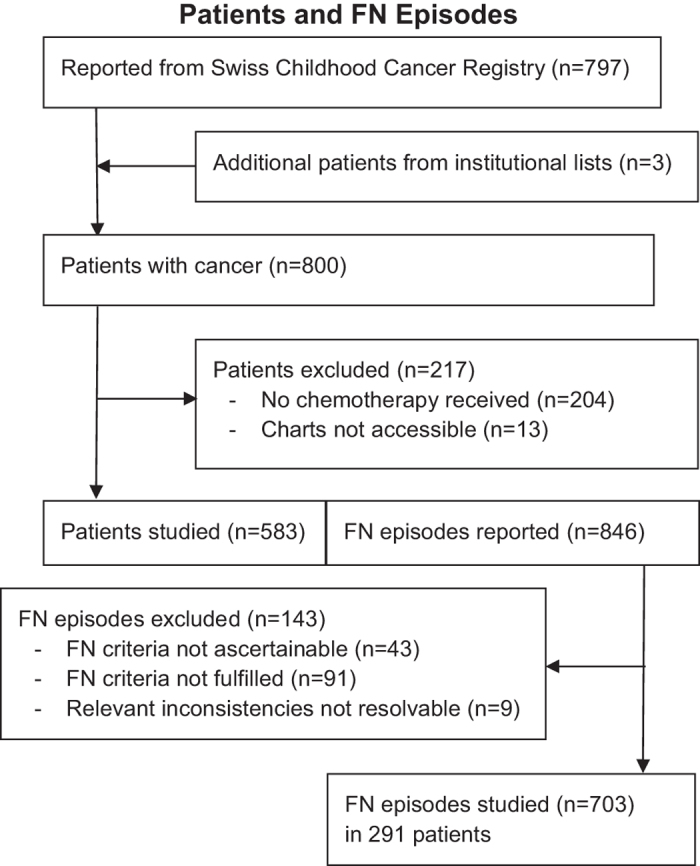
Flowsheet of patients and FN episodes.

From 1993 to 2012, 800 patients had been diagnosed with cancer up to the age of 17 years in Bern. Of these, 596 (75%) had received chemotherapy. Charts were not accessible in 13 (2%). In the remaining 583 (98% of 596) patients, 846 FN episodes had been clinically diagnosed. FN criteria were not ascertainable in 43 (5%) and not fulfilled in 91 (11%) of these episodes, and relevant non-resolvable inconsistencies led to the exclusion of 9 (1%) episodes. The remaining 703 (83%) FN episodes were studied here. (Fig. 1).

In the 291 (50%) of the 583 patients with at least one FN episode included in this study, the median number of episodes of FN per patient was 2 (maximum, 9). The distribution of gender, age at diagnosis and of diagnostic groups did not change significantly over time regarding both patients and episodes of FN. Bacteremia was detected in 148 (21%), and SBI in 357 (51%) of the 703 FN episodes.

Simultaneously collected data on the risk to develop FN during chemotherapy from the 583 patients with cancer and accessible charts, have been published in *Scientific Data* before^23^.

These data can mainly be used (1) to study FN characteristics over time and between centers; (2) to study the association of FN characteristics with outcomes over time and between centers; (3) to derive corresponding CDRs for risk-adapted treatment of FN in pediatric patients; and (4) to externally validate CDRs derived from other datasets.

## Methods

### Study design

A retrospective, single site cohort study covering two decades, from 1993 to 2012, was performed at the Division of Pediatric Hematology and Oncology, Department of Pediatrics, Inselspital, Bern University Hospital, University of Bern, Switzerland. All information was retrospectively extracted from patients charts as described^23,24^.

This study was approved by the Institutional Review Board (Direktion Lehre und Forschung, Inselspital Bern; registration number, 13-06-11; last update, April 02, 2014), including waiver of informed consent. On June 30, 2014, data were fully anonymized before analysis, in order to comply with the requests of the new Swiss Federal Law on Human Research.

Corresponding data on the risk to develop FN during chemotherapy have been published elsewhere^23,25^.

### Patients

All children and adolescents diagnosed with cancer (including Langerhans cell histiocytosis and all tumors of the central nervous system) and treated with chemotherapy were eligible. Patients were primarily identified via the Swiss Childhood Cancer Registry (SCCR)^26^. In order to reduce recruitment bias, clinically used patient lists and databases of previously published studies^4,8,24,27,28^, were searched. This resulted in 3 additional patients not listed in the SCCR database (Fig. 1). Extensive plausibility checks performed by an experienced pediatric oncologist (RAA) detected relevant inconsistencies in 10 FN episodes. Because data were fully anonymized, these inconsistencies could be resolved in only one episode, while the 9 remaining episodes were deleted from the database (Fig. 1).

Age at cancer diagnosis was restricted to  ≤17 years. Multiple FN episodes per patients were allowed. Information on clinical characteristics and FN episodes including outcomes was extracted directly from patient charts.

### Treatment of cancer

Most patients were treated according to established international protocols. Clinical management regarding prophylaxis and treatment of FN essentially remained unchanged during the entire study period. Patients did not receive any antibiotic prophylaxis beyond prophylaxis against *Pneumocystis jirovecii* pneumonia with oral trimethoprim/sulfamethoxazole, which was replaced by inhaled pentamidine in selected patients^24^. Daily subcutaneous granulocyte colony-stimulating factor (G-CSF) was applied if requested by protocol^24^.

### Fever in neutropenia, definition and treatment

An FN episode was defined as fever in a patient with severe chemotherapy-induced neutropenia. Until July 8, 2007, fever was defined as an axillary temperature ≥38.5 °C persisting ≥2 h, or a single temperature ≥39.0 °C. Since July 9, 2007, fever was defined as single tympanic temperature ≥39.0 °C^27^. In the setting of rising temperatures, the different limits used for the different measurement methods have been shown to be comparable^29^. Severe neutropenia was defined as an absolute neutrophil count (ANC) <0.5 G/L^30^. FN episodes additionally diagnosed for clinical reasons at lower temperatures and/or with an ANC ≥ 0.5 G/L^31^ were excluded and not studied here (Fig. 1). Bacteremia was defined as any bacteria detected in blood culture from the beginning until the end of the FN episode.

The beginning of an FN episode was defined as the time point when the FN criteria were fulfilled. The end of this episode was defined as the time point when antibiotics were stopped, the patient was discharged, or chemotherapy was restarted, whichever occurred earlier. If the FN criteria were fulfilled again after this time point, this counted as a further FN episode. Correspondingly, multiple FN episodes per observation period were possible. Taken together with the stopping criteria mentioned above, FN episodes could thus end despite continued neutropenia, and second FN episodes could be diagnosed within one episode of neutropenia^23^. Recovery from neutropenia was assumed when the ANC was rising, there was no specific absolute ANC level defining recovery.

Routine management for patients with FN included emergency hospitalization and empirical broad-spectrum intravenous antimicrobial therapy, usually once daily ceftriaxone plus amikacin^32^. In the absence of documented infection, intravenous antibiotics were continued until resolution of fever for ≥ 48 hours and increasing leukocyte count or ANC. First-day step-down to oral outpatient treatment was performed only in the second decade studied in FN episodes with low risk of complications; first, in the experimental arm of a prospective interventional study from 2004 to 2007^10^, and thereafter within clinical routine from 2010 onwards^8,33^.

### Characteristics studied

A total of 29 characteristics available at FN diagnosis were recorded. First, there were 6 patient- and cancer-related characteristics: Number of FN episode counted from the beginning of the study, sex, age group at cancer diagnosis (0 to 3.99 years, 4 to 7.99 years, 8 to 11.99 years, and 12 to 16.99 years), diagnostic group of cancer, first versus later cancer, non-relapsed versus relapsed cancer.

Second, there were 8 characteristics related to patient history known at FN diagnosis: Time period of FN diagnosis, age group at FN diagnosis, bone marrow involvement (≥5% leukemic cells, or any malignant cells in solid tumors, determined at the start of the last parenteral chemotherapy preceding the specific FN episode), intensity of chemotherapy (Table 1), prophylaxis with G-CSF or GM-CSF, central venous access device, hospitalization before FN diagnosis, and potential triggers for fever (transfusion, cytarabine).

Third, there were 8 characteristics related to findings on clinical examination at FN diagnosis: highest temperature reported or measured, general health condition, chills, oral mucositis, comorbidities requiring hospitalization independently from fever, clinical signs of bacterial infection, clinical signs of viral infection, and details regarding clinical signs of infection, if applicable.

Finally, there were 7 laboratory characteristics, all determined in peripheral blood at FN diagnosis: hemoglobin, leukocyte count, ANC, absolute monocyte count, absolute phagocyte count, thrombocyte count, and C-reactive protein (CRP).

### Outcomes studied

In total, 28 outcomes were recorded. First, 8 outcomes described treatment of FN: Choice of empirical antibiotics^32^, switching of intravenous antibiotics for any reason (step down from intravenous to oral did not count as switching), duration of intravenous antibiotics, duration of oral antibiotics, duration of intravenous antifungal therapy, intensive care unit (ICU) treatment for diagnostics or for organ support^34^, duration of ICU treatment, and duration of hospitalization for FN.

Second, 6 outcomes described microbiologically infections (MDI): Bacteremia, defined as any bacteria detected in blood culture before recovery from severe neutropenia (irrespective of the bacterial species^8^), bacterial species detected in blood, microbiologically defined bacterial infection other than bacteremia (irrespective of localization of infection or detection method), microbiologically defined fungal infection (irrespective of localization of infection or detection method), microbiologically defined viral infection (irrespective of localization of infection or detection method), and MDI of any kind^35^.

Third, 4 outcomes described clinically defined infections: Radiologically confirmed pneumonia, potentially life-threatening complication (PLTC) of infection as judged by the treating physician^8^, death from infection during neutropenia (outside palliative situations)^20^, and details describing the infection.

Fourth, in order to facilitate analysis, 4 additional clinical composite outcomes were defined: A serious medical complication (SMC) was defined as death from infection, ICU treatment, or PLTC^8^; an adverse event (AE) was defined as MDI, SMC, or radiologically confirmed pneumonia^8^; a severe bacterial infection (SBI) was defined as death from infection, positive bacterial culture of normally sterile body fluids (including bacteremia), radiologically confirmed pneumonia, clinically unequivocal diagnosis of a bacterial infection as judged by the treating physician or serum CRP >150 mg/L at FN diagnosis^8^; and unexplained fever was defined as FN without clinical or microbiological evidence of infection^35^.

Finally, 6 discharge-related outcomes were defined: Discharge with versus without antibiotics (prophylactic antibiotics not included), absolute leukocyte, neutrophil, monocyte and phagocyte counts at discharge, and rehospitalization because of fever or complications within 7 days from discharge.

Considering the set of 12 FN core outcomes recently proposed in a consensus statement of an international panel of FN experts^20^, 3 outcomes (MDI, unexplained fever and infection related mortality) can be assessed with these data without modification. Three further outcomes (bacteremia, transfer to ICU and SMC) can be assessed with modifications, while the 6 remaining outcomes of the proposed set (clinically documented infection, sepsis, severe sepsis, septic shock, all cause 30-days mortality, and relapse of primary infection) are not assessable here because of missing information.

### Data classification

In order not to compromise the irreversibility of anonymization, periods of 4 years were used for age groups both at cancer diagnosis and at FN diagnosis (4 periods: 0 to 3.99 years, 4 to 7.99 years, 8 to 11.99 years, and 12 to 16.99 years), and for time periods of FN diagnosis (5 periods: 1993 to 1996, 1997 to 2000, 2001 to 2004, 2005 to 2008, and 2009 to 2012).

Chemotherapy was classified into 4 levels of myelosuppressive intensity according to the expected duration of severe neutropenia as described^24,28^, which is an extension of an earlier model using only 2 levels^36^ (Table 1)^23^. This classification does not cover the additional risk of infection due to new therapeutic agents like rituximab that do not lead to relevant neutropenia.

### Code availability

This study did not use any computer codes to generate the dataset. Microsoft Excel was used to enter, store and quality check the collected data.

## Data Records

A single data record resulted from this study. It contains information of characteristics and outcomes on the 703 FN episodes periods studied in these 291 patients (File 17C.Characteristics_FN_Episodes.csv. Data Citation 1) (Table 2).

Information on patients at time of study entry can be extracted from this file by selecting the first FN episode per patient (EPI.PER.PAT = 1).

Detailed information on variable specification is included in a readme file (File 17C.Characteristics_FN_Episodes.ReadMe.csv. Data Citation 1).

## Technical Validation

Data was retrieved from patients charts into a paper case report form (File 17C_CRF.pdf. Data Citation 1) by different persons, i.e., co-investigators, medical students, and research assistants, all instructed by the senior author (RAA). The information of these CRF’s was then entered into a spreadsheet, without double-checking.

### Reduction of recruitment bias

Patients were primarily identified via the Swiss Childhood Cancer Registry^26^. In order to reduce recruitment bias, information on patients was complemented by clinically used institutional patients lists for the entire period, and patient lists from earlier research projects for a part of the period covered here^4,8,24,27,28^ (Fig. 1).

### Increasing reliability of information on FN episodes

A simple restricted definition of FN episodes, based on verifiable quantitative information both on fever and on neutropenia was used as described^23^. Correspondingly, FN episodes additionally diagnosed clinically when fever and or neutropenia limits had not been reached^31^, or when these limits were not ascertainable, were excluded.

A simple definition of bacteremia was used as well, without the need of partly subjective judgment if detection of a common commensal in a blood culture should be considered as infection, i.e., bacteremia, or as contamination.

In case of inconsistencies or unclear information found in the charts, the person retrieving data from charts called the senior author, an experienced pediatric haematologist-oncologist (RAA), to consult patient charts again and to resolve these questions. In the majority of relevant inconsistencies detected in the database only after irreversible anonymization, however, these could not be resolved any more, which led to the exclusion of the respective FN episode (Fig. 1).

## Additional information

**How to cite this article**: Zermatten, M. G. *et al*. Episodes of fever in neutropenia in pediatric patients with cancer in Bern, Switzerland, 1993–2012. *Sci. Data*. 6:180304 doi: 10.1038/sdata.2018.304 (2019).

**Publisher’s note**: Springer Nature remains neutral with regard to jurisdictional claims in published maps and institutional affiliations.

## Supplementary Material



## Figures and Tables

**Table 1:** Myelosuppressive intensity of chemotherapy.

Level	Intensity	Expected duration of severe neutropenia
1	Minimally myelosuppressive	None expected
2	Briefly myelosuppressive	≤10 days
3	Strongly myelosuppressive	>10 days
4	Myeloablative	Hematopoietic stem cell recovery required
Modified from refs^24^ and^28^, plus ref.^31^ regarding the distinction of level 2 versus 3.		

**Table 2:** Overview.

Observations	Time covered	Center	Data source	Data
Patients	1993 to 2012^a^	Bern^a^	Swiss Childhood Cancer Registry^26^ Institutional patient lists	17C.Characteristics_FN_Episodes.csv [EPI.PER.PAT = 1]
FN Episodes	1993 to 2012^a^	Bern^a^	Chart records	17C.Characteristics_FN_Episodes.csv

^a^Identical for both patients and FN Episodes.
